# Glucagon-Like Peptide-1, Diabetes, and Cognitive Decline: Possible Pathophysiological Links and Therapeutic Opportunities

**DOI:** 10.1155/2011/281674

**Published:** 2011-06-01

**Authors:** Enrico Mossello, Elena Ballini, Marta Boncinelli, Matteo Monami, Giuseppe Lonetto, Anna Maria Mello, Francesca Tarantini, Samuele Baldasseroni, Edoardo Mannucci, Niccolò Marchionni

**Affiliations:** Unit of Gerontology and Geriatric Medicine, Department of Critical Care Medicine and Surgery, University of Florence and Careggi Teaching Hospital, 50121 Florence, Italy

## Abstract

Metabolic and neurodegenerative disorders have a growing prevalence in Western countries. Available epidemiologic and neurobiological evidences support the existence of a pathophysiological link between these conditions. Glucagon-like peptide 1 (GLP-1), whose activity is reduced in insulin resistance, has been implicated in central nervous system function, including cognition, synaptic plasticity, and neurogenesis. We review the experimental researches suggesting that GLP-1 dysfunction might be a mediating factor between Type 2 diabetes mellitus (T2DM) and neurodegeneration. Drug treatments enhancing GLP-1 activity hold out hope for treatment and prevention of Alzheimer's disease (AD) and cognitive decline.

## 1. Insulin Resistance and Cognitive Decline

During the last years, Alzheimer's disease (AD) and clinical syndromes associated to insulin resistance have shown an ever-increasing prevalence in Western countries. These conditions pose a great threat to present and future population's health and represent two of the main causes of disability and health expenditures. Several research lines during the last decade have suggested an association among Type 2 diabetes mellitus (T2DM), insulin resistance, and cognitive decline, both in cross-sectional and in longitudinal studies. 

Cross-sectional studies have found that older subjects with T2DM on average show a poorer cognitive performance than age-matched controls [1]. This association seems independent of other vascular risk factors and is attributable not only to a greater extent of white matter lesions but also to a more severe cortical atrophy [2], especially in temporo-mesial areas (hippocampus, amygdala) [3]. Moreover insulin resistance is associated to a worse cognitive performance in nondiabetic subjects too [4]. On the other hand cross-sectional studies have observed a significant association of dementia, AD in particular, with T2DM [5] and insulin resistance [6]. 

Also several longitudinal studies have observed an association of T2DM with dementia risk over years [7]. Moreover it has been observed that older nondiabetic subjects with metabolic syndrome and increased level of inflammatory markers have an increased risk of subsequent cognitive decline [8]. Recently published data have shown that, among nondiabetic nondemented older subjects, insulin resistance is associated with AD incidence after a few years [9]. In keeping with this observation, insulin resistance has been associated recently with a greater extent of AD-like neuropathology at autopsy [10]. Therefore it is plausible that, among older subjects with asymptomatic AD, a coexistent impairment of insulin metabolism can hasten symptoms expression. 

This association might be linked to different biological mechanisms, first of all the presence of brain insulin resistance. In fact brain insulin receptor activity might have several neuroprotective effects, via PI3K (phosphatidyl-inositol-3-kinase)/Akt and ERK1/2 signalling pathways [11, 12]: decreased inflammation and apoptosis, increased synaptic plasticity, and inhibition of glycogen synthase kinase-(GSK-)3, with subsequent decreased tau phosphorylation, which is a hallmark of AD neuropathology. It has been shown in vitro that insulin receptor activation is able to decrease synaptic binding sites through which amyloid oligomers produce their toxic activity, with resulting reduction of oxidative stress and dendritic spines loss [13]. Moreover postmortem analyses of AD patients brain have shown an impairment of insulin and IGF-1 receptors signalling, especially evident in neurons with neurofibrillary tangles, suggesting that degenerating neurons are resistant to insulin/IGF-1 action [14]. Some authors have even proposed the existence of a “Type 3 diabetes mellitus”, limited to central nervous system (CNS), as a cause for AD, as they were able to produce an AD-like neurodegeneration in a mouse model after intracerebroventricular injection of streptozotocin, inducing a depletion of CSF insulin without any change in peripheral insulin metabolism [15]. This hypothesis is supported by a pilot study, which has shown a significant cognitive improvement after intranasal insulin, without change of peripheral glucose metabolism [16].

On the other hand, experimental data have associated peripheral insulin resistance with reduced insulin activity inside the CNS, due to a reduced hormone transport through the blood-brain barrier [17], and with increased brain A*β* production in murine models of AD [18]. Moreover in studies of normal subjects with euglycemic clamp, the infusion of high insulin doses, mimicking insulin resistance, raises A*β*-42 levels, probably due to a reduced catabolism, and CNS inflammatory markers [19].

## 2. Metabolic Effects of GLP-1, T2DM, and the “Gut-to-Brain” Axis

Glucagon-like peptide-1 (GLP-1), a member of the incretins family, is a 30-aminoacid peptide, which is derived from pre-proglucagon molecule and is secreted by intestinal endocrine epithelial L-cells. It is the most potent stimulator of oral glucose-induced insulin secretion, it is released in response to meal intake and is rapidly metabolized and inactivated by dipeptidyl-peptidase-4 [20]. GLP-1 transmembrane receptor (GLP-1R) is a G-protein-coupled receptor and is expressed not only in pancreatic islets, but also in gastrointestinal tract, kidney, lung, vascular system, heart, and brain [21]. 

GLP-1R activation stimulates adenylate cyclase, with formation of cyclic adenosine monophosphate (cAMP) and subsequent phosphorylation of protein kinase A; moreover it activates PI3-kinase pathway, with downstream activation of Akt kinase, MAP-kinases, and src-kinases [22, 23]. Via these pathways, GLP-1 stimulates pancreatic *β*-cells, activating insulin secretion and inducing insulin gene expression [21]; moreover it has been shown that GLP-1 stimulates proliferation and differentiation, and reduces apoptosis of *β*-cells [23]. It seems interesting that, at least in pancreatic islets, GLP-1 activity seems synergic with insulin action in promoting *β*-cell survival [24].

Beyond its main activity, GLP-1 reduces plasma glucagon, inhibits gastrointestinal motility, and promotes satiety, reducing food intake [21]. Moreover it has a wide range of functions on glucose metabolism and cardiovascular system. In fact it improves insulin sensitivity, reduces appetite, modulates heart rate and blood pressure, reduces vascular tone, ameliorates endothelial function, and increases myocardial contractility, with preliminary data suggesting clinical benefit in heart failure [25]. 

It has been known for many years that T2DM is characterized by a severely reduced incretin effect, defined as the difference between insulin responses to oral and intravenous glucose administration [26]. Reduced GLP-1 levels have been observed after a mixed meal in Type 2 diabetes compared with controls [27], with a marked reduction especially of the late-phase response [28]. Moreover an altered GLP-1 response both to mixed meal [29] and to oral glucose load [30] has been observed in insulin resistance.

It has been proven that at least part of the metabolic effect of GLP-1 is mediated by CNS [31]. In fact brain GLP-1R are partly responsible not only for food intake control, but also for control of glucose homeostasis, with coordinate actions on pancreas and liver [32]. It has been observed in mice that GLP-1 secreted into the hepatoportal vein increases the firing rate of the vagus nerve, sending signals to the brainstem nucleus of the tractus solitarius, which on the other hand releases GLP-1 in hypothalamic regions, inducing reflex insulin secretion and muscle glycogen synthesis [33]. These data support the existence of a “gut-to-brain axis”, with a central role of GLP-1 released both by intestinal cells and by neurons, involved in the regulation of systemic glucose metabolism, whose activity seems to be blunted in high-fat fed, insulin-resistant mice [33].

On the other hand, it has been hypothesized that GLP-1 can influence brain metabolism. In fact a small human study with FDG-PET (positron emission tomography with 18-fluorodeoxyglucose) has shown a possible effect of GLP-1 on brain glucose metabolism. In this study GLP-1 infusion in normoglycemic conditions reduced glucose transport across blood-brain barrier in specific brain areas while a trend of decrease of cerebral metabolic rate was also observed, thus maintaining brain glucose concentration unchanged. This observation leads the authors to hypothesize that GLP-1 may exert a neuroprotective effect by limiting intracerebral glucose fluctuation in postprandial periods, when plasma glucose is increased [34].

## 3. GLP-1, Neuroprotection, and Alzheimer's Disease

Beyond its metabolic role, several studies have clarified a role of GLP-1 in CNS function. Experimental studies have identified a widespread expression of GLP-1R across a large number of rat brain regions, not directly involved in metabolic control, including hippocampus, thalamus, striatum, substantia nigra, amygdala, nucleus basalis Meynert, subventricular zone, and temporal cortex [35]. GLP-1R expression has been observed in specific cellular subtypes which are crucial for memory and learning functions, including pyramidal neurons of CA region and granule cells of dentate gyrus in hippocampus, and in large neocortical neurons [36]. Other authors have observed GLP-1R expression also on glial cells (microglia and astrocytes), proposing a role for them as modulators of CNS inflammation [37]. 

The neurotrophic effect of GLP-1R has been strongly suggested by studies of mice knockout (KO) for GLP-1R, which show an impairment of contextual memory, as assessed by the passive avoidance test, which measures the ability of the animal to learn and remember that an instinctive behavior causes a punishment. Memory impairment of KO mice was reversible after GLP1-R gene DNA transfer with a viral vector [38]. These data were confirmed in a subsequent study of cognitive functions in a GLP-1R KO mouse model: a reduced recognition memory and spatial memory has been shown while other behavioural parameters, including exploration and anxiety, were unchanged. Interestingly a neurophysiological study of hippocampus CA1 area mice showed a severe impairment of long-term potentiation, which is the synaptic process associated to consolidation of long-term memory [39]. 

Adding to these observations, it has been demonstrated that GLP-1 analogues, which have greater metabolic stability than the native molecule, also cross blood-brain barrier when administered peripherally [40]. As only small amounts of native GLP-1 reach CNS if peripherally administered, due to rapid catabolism, much of the pharmacologic research has focused on analogues of the molecule, which are more resistant to degradation, while retaining the stimulatory effect on GLP-1R. 

Several experimental evidences have demonstrated a neuroprotective role for GLP-1 and its analogues. In cultured rat pheochromocytoma cells, some authors observed that GLP-1 and exendin-4, (*Ex-4*) a long-acting GLP-1 analogue, stimulated neurite outgrowth in a similar fashion to nerve-growth-factor (NGF). Besides, *Ex-4* was able to augment NGF-induced neuronal differentiation, and apparently attenuated neural degeneration following NGF withdrawal [41]. Other authors confirmed these data on cultured neural cells, finding that GLP-1 exposure protected cells from death promoted by NGF deprivation, by suppressing the proapoptotic protein Bim (Bcl-2 interacting mediator of cell death) [42]. 

Part of the neuroprotective effect of GLP-1R agonists is probably related to reduced neuronal damage due to amyloid metabolism. In fact *Ex-4* has been shown to reduce the synthesis of amyloidogenic A*β* fragment and to protect cells from *β*-amyloid toxicity in cultured neural cells [43]. Moreover intracerebroventricular injection of GLP-1 or *Ex-4* has been shown to decrease levels of brain amyloid fragment in control mice [44].

The efficacy of peripherally administered GLP-1 analogues has been shown also in experimental models. In normal adult rats *Ex-4* improves hippocampus-based cognitive performance, namely, spatial learning and working memory, as assessed by the “radial arm maze,” which allows the measurement of the time necessary for the animal to find food, placed at the end of several equidistantly spaced arms, which radiate form a central platform [45]. In the same paper the repeated administration of *Ex-4* was effective in ameliorating mood and reducing hopelessness, as measured by the immobility time in the “forced swim test,” during which animals are forced to swim in a cylinder filled with water, from which they cannot escape [45]. 

The previously mentioned behavioural effects are paralleled by several histochemical changes observed “ex vivo.” Intraperitoneal administration of *Ex-4* has increased both the number of proliferating cells and the expression of neuronal differentiation markers in adult rat hippocampus and in subventricular zone [45, 46]. 

A neuroprotective effect of *Ex-4* has been observed recently in experimental models of neurodegeneration. In the triple transgenic AD-mouse, which is an experimental model of the human disease, the induction of diabetes with streptozotocin was associated with an increase of *β*-amyloid brain load, consistently with evidence linking T2DM with AD neuropathology, and subcutaneous administration of *Ex-4* prevented this increase [43]. The results of this study suggests that *Ex-4* may have a therapeutic role in AD, alone or with T2DM. Moreover in an animal model of Parkinson's disease, in which *Ex-4* was able to increase the number of dopaminergic neurons in the substantia nigra, a contemporary reduction of extrapyramidal signs was observed [46]. 

Another long-acting GLP-1 analogue used for T2DM, liraglutide (*Lir*), is able to cross the blood-brain barrier [47], and has shown neuroprotective effects in experimental models. This is also the case for other GLP-1 mimetics, Asp(7)GLP-1, N-glyc-GLP-1, and Pro(9)GLP-1, which, like *Lir*, are able to increase synaptic plasticity, measured as long-term potentation in CA1 hippocampal region of rats [47].

Both *Ex-4 *and *Lir *were recently tested for their neuroprotective effect in mouse models of T2DM. In this study GLP-1 analogues were injected subcutaneously to three mouse models of diabetes (ob/ob mice, db/db mice, and high-fat-diet-fed mice). At the histochemical analysis a greater number of proliferating neurons in hippocampal dentate gyrus was found in diabetic mice compared with nondiabetic controls, and this number was further enhanced by both drugs [48]. The increased neurogenesis in T2DM models was interpreted by the authors as a response to increased brain cell death which is associated with the disease; this compensatory process would be supported by GLP-1 mimetics. This interpretation is supported by another paper published by the same authors, regarding the cognitive effect of *Lir* in the mouse model of high-fat-diet-induced obesity. In parallel with metabolic changes (weight loss, increased glucose tolerance), mice treated with *Lir* subcutaneous injections showed an improvement of learning and memory ability, assessed with “object recognition test.” The test measures the extent of exploratory activity of a previously presented object, which is expected to be lower in comparison with newly presented objects, and is therefore considered a measure of recognition memory. Furthermore, *Lir* reduced negative effects of high-fat diet on hippocampal long-term potentation [49]. 

Another analogue of GLP-1, Val(8)-GLP-1(7-36), was studied in rats, and its intracerebroventricular injection reversed the impairment of spatial memory induced by injection of *β*-amyloid fragment A*β*1–40. Moreover pretreatment with Val8-glucagon-like peptide-1 prevented the impairment of hippocampal long-term potentiation that is induced by the presence of A*β*1–40 [50]. These data are consistent with a different research, performed with the same molecule, on AD-like mice with a double mutation of amyloid precursor protein (APP) and presenilin 1 (PS1). A beneficial effect was observed on long-term potentiation, both in young (9 months) and in older animals (18 months) while *β*-amyloid plaques and inflammatory microglia activation was unchanged in treated animals [51]. These data support the hypothesis that GLP-1R agonists might partly prevent toxic effect of *β*-amyloid deposition, with obvious interest for possible AD treatment.

With the background of the previously discussed preclinical data, *Ex-4* is now being studied as a treatment for AD and PD in Phase 2 studies (see http://www.clinicaltrials.gov/, NCT01255163 and NCT01174810). Of notice, the hypoglycemic effect of GLP-1 analogues in normoglycemic subjects seems minimal [52, 53] and should not constitute a major concern for the treatment of nondiabetic subjects.

## 4. Conclusions

The available evidences strongly support the hypothesis that the observed association between insulin resistance/T2DM and cognitive decline/dementia is mediated not only by well-known vascular changes, but also by direct neurotoxic effect of glucose metabolism impairment.

Incretin activity, and GLP-1 in particular, which is reduced in insulin resistance conditions, represents a possible pathophysiological link between metabolism disorders and neurodegeneration. The reduction of GLP-1 levels, characteristic of T2DM, might be associated to a reduced neuroprotection, which seems particularly relevant for hippocampal regions [39], where AD neuropathology is most evident. It is tempting to speculate that GLP-1 and insulin have synergistic activity in promoting neuron survival, as it has been shown in pancreatic *β*-cells, both for native GLP-1 [24] and for GLP-1 synthetic analogues [54, 55]. Human studies evaluating the association between GLP-1 levels and cognitive function, controlling for insulin resistance status, are needed to support the hypothesis of a direct neuroprotective effect of incretins. 

GLP-1 analogues has been shown to enhance cognitive function in control animals [38, 45], to prevent cognitive impairment in models of T2DM [48, 49], and to counteract *β*-amyloid toxicity in models of AD [43, 51]. These experimental data support the effort of testing such molecules both for the prevention of cognitive decline in T2DM and for treatment of AD patients in randomized controlled trials.
